# Flexibility of *in vitro* cortical circuits influences resilience from microtrauma

**DOI:** 10.3389/fncel.2022.991740

**Published:** 2022-12-16

**Authors:** Modupe A. Adegoke, Olivia Teter, David F. Meaney

**Affiliations:** ^1^Department of Bioengineering, School of Engineering and Applied Sciences, University of Pennsylvania, Philadelphia, PA, United States; ^2^Department of Neurosurgery, Penn Center for Brain Injury and Repair, Perelman School of Medicine, University of Pennsylvania, Philadelphia, PA, United States

**Keywords:** traumatic brain injury, neuronal networks, connectivity, circuit scaling, susceptibility

## Abstract

**Background:**

Small clusters comprising hundreds to thousands of neurons are an important level of brain architecture that correlates single neuronal properties to fulfill brain function, but the specific mechanisms through which this scaling occurs are not well understood. In this study, we developed an *in vitro* experimental platform of small neuronal circuits (islands) to probe the importance of structural properties for their development, physiology, and response to microtrauma.

**Methods:**

Primary cortical neurons were plated on a substrate patterned to promote attachment in clusters of hundreds of cells (islands), transduced with GCaMP6f, allowed to mature until 10–13 days *in vitro* (DIV), and monitored with Ca^2+^ as a non-invasive proxy for electrical activity. We adjusted two structural factors–island size and cellular density–to evaluate their role in guiding spontaneous activity and network formation in neuronal islands.

**Results:**

We found cellular density, but not island size, regulates of circuit activity and network function in this system. Low cellular density islands can achieve many states of activity, while high cellular density biases islands towards a limited regime characterized by low rates of activity and high synchronization, a property we summarized as “flexibility.” The injury severity required for an island to lose activity in 50% of its population was significantly higher in low-density, high flexibility islands.

**Conclusion:**

Together, these studies demonstrate flexible living cortical circuits are more resilient to microtrauma, providing the first evidence that initial circuit state may be a key factor to consider when evaluating the consequences of trauma to the cortex.

## Introduction

The nervous system is one of the most complex organs in the human body, with layers of architecture spanning from a few microns to several meters (Yuste, 2015). While allowing for very diverse and complex function, the multi-scale architecture of the brain also makes it susceptible to many diseases (Aerts et al., 2016; Dulla et al., 2016), as alterations in function at the lower cellular and molecular level can compound and spread to give rise to high-level cognitive dysfunction (Seeley et al., 2009; Fornito et al., 2015). One such condition, traumatic brain injury (TBI), affects many of the levels of architecture intrinsic to the brain (Wolf and Koch, 2016; Kenzie et al., 2017), and it can have long-lasting sequelae potentially evolving into neurodegenerative disease (Hoskison et al., 2009; Gupta and Sen, 2016; Hay et al., 2016; Taylor et al., 2017; Tagge et al., 2018).

Top-down approaches for determining alterations in brain architecture often use high-resolution imaging studies to capture full brain activity and determine areas of the brain that are activated simultaneously during cognition, producing functional connection maps of areas across the entire brain. The term brain “connectome” has emerged as a way to characterize the underlying structural and functional networks of the brain (Behrens and Sporns, 2012; Leergaard et al., 2012; Fornito et al., 2015; Petersen and Sporns, 2015), and network analysis tools provide an effective way to investigate brain dynamics across scales with respect to information integration, segregation, distribution into modules and the function of hub nodes. One advantage of this approach is that it promoted scaling of brain architectures across species, or from small to large brain areas within an individual brain, establishing a path to connect organizational levels of the brain with cognitive function (Betzel et al., 2016; Bassett and Sporns, 2017; Betzel and Bassett, 2017; Jeub et al., 2018).

Using this top-down approach, drawing relationships between brain subnetworks and brain function offer an attractive perspective for understanding neurological diseases, and recent reviews have highlighted the importance of topological location of traumatic injury on the resulting outcomes in TBI patients. Recent work shows that specific brain areas are more susceptible (Aerts et al., 2016), and the broad effects of injury on brain networks can ultimately be correlated to cognitive outcome (Sharp et al., 2014). Additionally, functional MRI studies in TBI patients report long-lasting altered connectivity, disconnection within the default mode network and impaired modular structure (Douw et al., 2010; Caeyenberghs et al., 2012, 2013, 2014; Pandit et al., 2013; Han et al., 2014; Power et al., 2014; Fagerholm et al., 2015), features that can have a much broader impact on several aspects of cognition. These alterations in functional connections are also matched with broad disruptions of networks mapped with diffusion tensor imaging (DTI) (Yuan et al., 2015), electroencephalography (EEG) (Chennu et al., 2017), magnetoencephalography (MEG) (Castellanos et al., 2011) and functional near infrared spectroscopy (FNIRS) (Urban et al., 2015). Most importantly, long-term imaging follow-up studies of TBI patients point to an incredible ability of the circuitry to remodel and a direct correlation between this remodeling capacity and cognitive outcome (Bashir et al., 2012; Kou and Iraji, 2014; Sierra et al., 2015; Herbet et al., 2016).

Recent computational studies are using alterations in mesoscale components–e.g., a loss of nodes or the removal of edges connecting nodes–to identify important aspects of the impairment and remodeling process (Fasoli et al., 2016; Ocker et al., 2017). For example, some areas within the brain connectome may be more significant for impairment, e.g., lesions in cortical midline structures effect network structure more than lesions isolated to the motor cortex (Alstott et al., 2009). Interestingly, it is not only the deletion of nodes but their re-assembly, through neurogenesis, that can lead to a transient loss and recovery of global efficiency (Rubinov et al., 2009) that can be predicted with graph theory metrics (Young et al., 2000; Váša et al., 2015).

One critical missing component among existing studies is how nodes within either a structural or functional wiring of the brain can be impaired, and how this nodal impairment is influenced by the local topology of the microcircuit within the node. Certainly, dissociated cultures of neurons and glia will form spontaneous connections and microcircuits *in vitro*, and studying the resulting spontaneous activity patterns have led to critical insights into how bursts within the network are regulated by the connection density, balance among excitatory and inhibitory neurons, and the local synaptic properties (Mazzoni et al., 2007; Penn et al., 2016; Yamamoto et al., 2016; Huang et al., 2017). However, less common are studies to precisely engineer microcircuit properties and study how neuronal density, connectivity, and microcircuit size can influence activity.

*In vitro* dissociated cultures of neurons have greatly increased our understanding of both single-cell and circuit-level effects following injury. Dissociated culture and slice studies parsing out post-injury function have shown that even in the absence of neuronal death, injury disrupts circuit function on many levels, including activity rate, synchronization and excitatory/inhibitory balance (Cohen et al., 2007; Yu et al., 2009; Kutzing et al., 2011; Johnson et al., 2014; Kang et al., 2015). After trauma, we now know that there is a general breakdown of the network into multiple clusters (Patel et al., 2012) and loss of small world topology and network efficiency (Srinivas et al., 2007). However, since they generally rely on imaging techniques which only sample a small area of the larger culture, one major limitation of *in vitro* studies is that they lack the information about adjacent connected areas which influence properties of the observed area.

To address this limitation, several models of small neuronal cultures (microcultures) have been developed. Early studies of neuronal circuits developed the methods to culture small neuronal clusters and demonstrated their viability for studies of structure and function in small circuits of various neuronal cell types (Segal and Furshpan, 1990; Ranieri et al., 1994; Mennerick et al., 1995; Chang et al., 2003; Allen, 2007; Zeng et al., 2007; Ruiz et al., 2008; Sgro et al., 2011; Jang and Nam, 2012). Since then, micro-cultures have been used to parse out hippocampal synchronization (Cohen and Segal, 2011), high-throughput drug testing (Kang et al., 2009) or the emergence of bursting and assortative connectivity between connected neuronal clusters (Bisio et al., 2014; Teller et al., 2014). To our knowledge, this experimental platform has not been used in the context of injury.

In this study, we aimed to understand if features of the microcircuit features influence how traumatic injury affects circuit performance. Specifically, we aimed to generate and characterize small neuronal circuits and probe the importance of their structural properties to functional outcome, specifically focusing on resilience to injury. First, we developed an *in vitro* experimental platform for small neuronal circuits (islands) which allows us to address questions related to network development, physiology, and pathology in response to external perturbations. We characterized the influence of two easily adjustable structural factors (island size and cellular density) in guiding spontaneous activity and network formation in neuronal islands, and probed the activity response to a broad pharmacological alteration of the excitatory/inhibitory balance in the circuit. Finally, we used our understanding of how island activity is shaped by structural parameters to derive fundamental principles related to circuit resilience to a single-cell ablation injury model.

## Materials and methods

We investigated the role of two design parameters, island surface area (size) and cellular density, in guiding neuronal island activity state and response to pharmacological and mechanical alterations of circuit integrity.

### Neuronal island fabrication

**FIGURE 1:**
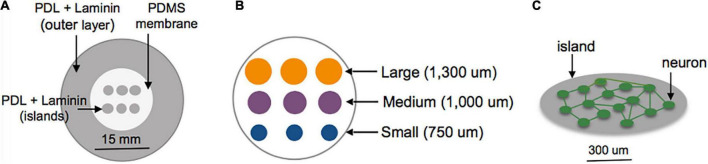
Schematic of island fabrication. **(A)** A thin, patterned polydimethylsiloxane (PDMS) membrane was used to restrict adsorption of PDL and laminin to circular regions of the center glass area of MatTek dishes. The outer region of the dish was left uncovered to encourage diffuse attachment of neurons into a feeder layer. **(B)** Detailed diagram of the center glass area of a MatTek dish, patterned with a grid of islands varying in size from 750 to 1,300 um. **(C)** Example of patterned island with neurons allowed to develop spontaneous connections within the island.

Three days prior to plating, the center surface of glass-bottomed MatTek dishes (MatTek Corporation, Ashland, MA, USA) was covered with a polydimethylsiloxane (PDMS) membrane patterned with grids of circles to selectively expose only the grid region of the glass area in the MatTek dishes (Figure 1A). Two configurations were tested: Islands of similar cellular density but varying sizes (750–1,300 um Figure 1B); islands of similar size but varying plating density (50–400 cells/mm^2^). The outer region of the dish was left uncovered by the PDMS membrane. Sequential adsorption of Poly-D-lysine (PDL) (1 mg/ml), laminin (50 ug/ml) and plating media (Minimum Essential Media, Gibco; heat-inactivated horse serum, Sigma, 10% by volume; Penn Strep, Gibco, 1% by volume; D-glucose, Sigma, 2.5 mg/ml) was performed over the course of 3 days. The PDMS inserts were removed immediately before plating of cortical neuronal cells harvested from embryos of timed pregnant Sprague-Dawley rats at day 18. Cells were dissociated, re-suspended, plated on the patterned substrates at a density of 200,000 cells/ml (10,000 cells/mm^2^) in plating media over three isolations to form an interconnected neuronal network (Figure 1C). Following overnight adhesion, media was replaced with Neurobasal supplemented with B-27 Supplement and GlutaMAX (Gibco, 1×; Gibco, 1×) and cells were grown in a humidified incubator at 37°C and 5% CO_2_ until experiments were performed at 10–13 DIV. Our experience with this culturing technique shows that the cortical neurons are approximately 80% excitatory and 20% inhibitory, as indicated by VGAT staining (Patel et al., 2014). These are roughly consistent with *in vivo* measures of GAD67+ neurons in the developing mouse cortex. Experiments were repeated over a minimum of three isolations (Sahara et al., 2012).

### Immunocytochemistry

Preferential attachment of cells to the patterned areas was assessed at 14 DIV with immunocytochemistry after fixation with 4% Paraformaldehyde (PFA). Samples were permeabilized for 5 min at r. t. with a dilute Triton-X 100 in PBS solution (Thermo Scientific, 0.2%), blocked for 45 min at r.t. with bovine serum albumin (BSA)/ normal goal serum (NGS) solution (1% by mass, 2% by volume, respectively), and incubated at 4°C overnight with primary antibodies to laminin (Rb∼ laminin, Abcam Ab11575, 1:500) and MAP2, a neuron-specific cytoskeletal protein (Ms∼ MAP2, Millipore, Mab 3418, 1:5,000). Secondary antibodies conjugated to a fluorescent probe were used to stain the samples for 45 min at r.t. (Alexa Fluor, Gt∼Ms, 568 nm, 1:1,000; Alexa Fluor, Gt∼Rb, 633 nm, 1:1,000), as well as Hoechst (Life Technologies, Salt Lake City, Utah, 33342, 10 um/ml). Imaging was performed on a Nikon TE2000 confocal microscope equipped with a 4× objective (Plan Apo, N.A = 0.2).

### Calcium imaging

Seven days before initial imaging (at DIV 3), cells were transduced with the genetically encoded Ca^2+^ indicator GCamP6f on a neurons-specific promoter (AAV1.Syn, Penn Vector Core). Ca^2+^ transients were recorded as a non-invasive assessment of functional activity of both the individual cells, as well as of the full neuronal circuit. Imaging was performed on a Nikon Eclipse TE2000 confocal microscope equipped with a 488 nm excitation laser (Prairie Technologies, Bruker, Millerica, MA, USA, Aurora), a spinning disk confocal unit (CSU-10b, Solamere Technologies, Salt Lake City, Utah), a CCD camera (Photometrics CoolSNAP HQ^2^) and a 4× objective (Nikon, Plan Apo, N.A = 0.2), which allowed for monitoring of full islands. An environmental chamber set up around the microscope was used to maintain cultures at 37°C and 5% CO_2_ during imaging in pre-conditioned Neurobasal supplemented with B27 and GlutaMAX (Gibco, 1×; Gibco, 1×). Images of 520 × 696 pixels were acquired for 3 min at 20 fps for each condition tested and saved as .tiff stacks.

### Longitudinal assessment of activity

In islands of varying sizes, the emergence and development of activity was monitored with 3 min Ca^2+^ recordings at DIV 10 (early) and DIV 13 (late). One of each island size (small, medium, and large) was monitored in each dish. The same islands were followed between the early and late time point, and co-registration of the images as described below allowed for single-cell tracking over time.

### Microablation injury

**FIGURE 2:**
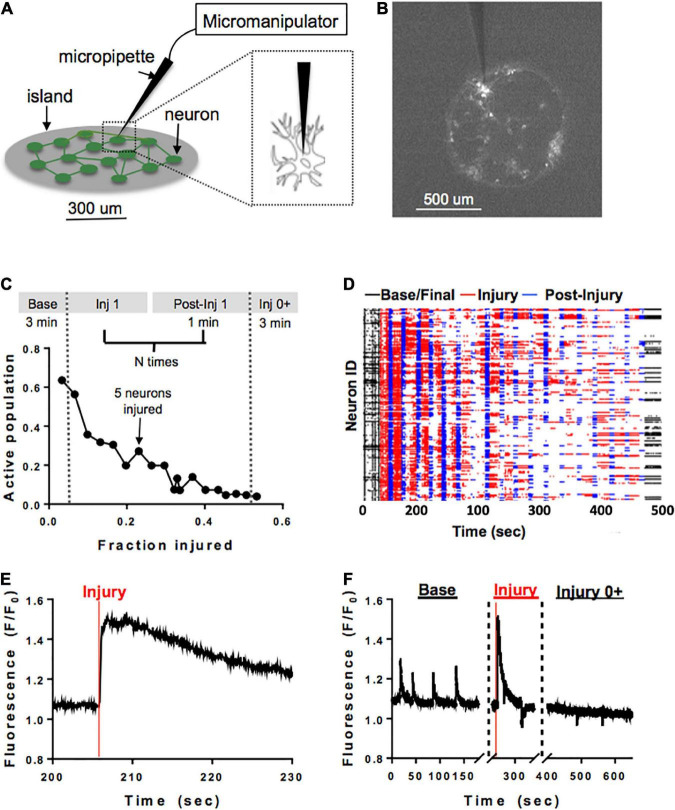
Microablation injury model. Schematic of microablation injury. **(A)** A micropipette tip (5–10 um diameter) attached to a FemtoJet device was momentarily brought into contact with individual neurons at a 90-degree angle. The individual neurons were physically impacted with the micropipette, leading to a transient mechanoporation of the cell and permanent deactivation. **(B)** Example of a small island under ×4 magnification. Each white dot represents a cell expressing GCaMP6f stimulated with 488 nm laser light. **(C)** Schematic of sequential injury protocol, where five neurons were chosen for microablation during each injury period, up to a total of 75% of an island’s population. **(D)** Ca^2+^ recordings from an island undergoing a sequential injury of 5 neurons at a time (red bands) and post-injury (blue bands) periods, in addition to an initial and final spontaneous activity recording (black bands). Partially synchronous spontaneous activity (black, left) was gradually reduced, and synchrony was lost in post-injury intervals (blue) as the level of injury was increased until activity was silenced by visual inspection. Few cells were still active in the final post-injury recording (black, right). **(E,F)** Zoom in on injury in a single neuron. The initial regular spontaneous Ca^2+^ activity in a neuron is interrupted by a large increase in intracellular Ca^2+^ as the micropipette is brought into contact with the cell, likely corresponding to transient mechanoporation, **(E)** followed by permanent inactivation in the subsequent recording session **(F)**.

A separate subset of neuronal islands of varying cellular densities was subjected to a model of single-cell mechanical injury which gradually reduced the number of neurons available in the circuit. Specifically, a FemtoJet microinjector device (Eppendorf) equipped with a glass pipette tip (5–10 um diameter) was used to physically impact the membrane of neurons (Figure 2A) while acquiring Ca^2+^ recordings as described before (Figure 2B). During each injury period, an overhead bright field lamp was turned on to better visualize the islands, five neurons were targeted with the injury device and 1 min post-injury Ca^2+^ recording were acquired (Figure 2C). Preliminary studies showed that it was not possible to compute functional connectivity of individual neurons without affecting neuronal viability. For that reason, neurons were randomly selected for mechanical ablation. Injuries were repeated until there were no active cells left in the island by visual inspection. Following the last mechanical ablation phase, networks were imaged continuously for 3 min to determine if some mechanically inactivated neurons resumed some activity. This resulted in a series of short injury/post-injury activity recordings, as well as two longer, 3 min baseline and final activity recordings (Figures 2D–F). In some cases, Controls matching in size and cellular density were exposed to the same illumination pattern for the same number of recordings, but the injury device was not brought in contact with the cells.

### Data analysis

A custom MATLAB analysis pipeline was developed to extract key information from the raw image stacks as described in Supplementary Figure 1.

Specifically, cell bodies were first identified as regions of interest (ROIs) from a maximum intensity projection of the binary image stack corresponding to each recording condition. The resulting binary mask was superimposed over the original image stack to extract fluorescence intensity over time from both the full field of view (full field fluorescence), as well as from the individual ROIs (single cell fluorescence). To facilitate the alignment of masks from all conditions corresponding to an island (registration), we developed a graphic user interface incorporating intensity-based automatic image registration using built-in functions in MATLAB (Image Processing Toolbox) and a user-driven registration algorithm. This ensured that the same population of neurons was accurately followed across conditions with single-cell resolution.

Single-cell fluorescence traces over time were background subtracted (F-F_*background)*_) and scaled to the interpolated single-cell baseline value (F/F_0_) to eliminate artificial sources of signal variability such as background noise, uneven illumination or variable expression levels of the virus. This resulted in single neuron activity traces with a constant baseline fluorescence level and superimposed Ca^2+^ transients. Calcium transients were automatically detected for each neuron using a custom-built peak detection algorithm correlating short fragments of the transient to a library of 128 representative spike waveforms (Patel et al., 2015). All transients detected were inspected for accuracy by a human evaluator. ROIs that did not show any detectable Ca^2+^ transients throughout any of the conditions recorded were eliminated as segmentation artifacts or dead cells.

**FIGURE 3:**
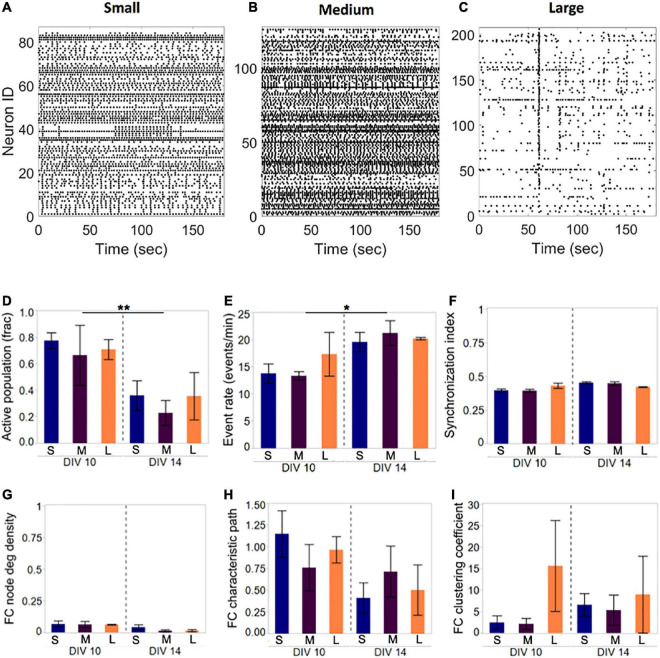
Activity and network development in neuronal islands of different sizes. **(A–C)** Representative examples of activity patterns in neuronal islands of varying size. Activity raster plots of islands ranging in surface area between 1.1 mm^2^ (small, panel **A**) to 1.5 mm^2^ (medium, panel **B**) to 2.2 mm^2^ (large, panel **C**). At 10 DIV, none of the activity or network metrics computed were significantly different between the three island size categories. Low synchronization index values (<0.5) and functional connectivity node density (<0.1) indicate sparsely correlated activity and connected networks. (**D–I**) By 14 DIV, the fraction of the neuronal population participating in spontaneous activity decreased uniformly in all island sizes, but activity rate increased significantly in the remaining active neurons (**D,E**). Developmental age did not significantly alter the synchronization level (**F**), nor the functional network structure as assessed by the three network metrics (**F,G**). Error bars indicate SEM. Significance level: *p < 0.05; **p < 0.01.

In addition to single-cell fluorescence, island-level activity was aggregated into activity raster plots (Figures 3A–C). Three metrics of activity were computed to characterize patterns of activity that emerge at the population level for each condition:

Active population–fraction of the neuronal population that is active at a given condition, calculated as the number of active cells over total cell number, ranging [0;1];

Event rate–average rate of activity for the active population, computed as the average event rate over all of the active cells in the circuit, as low as 0; synchronization index (SI)–global measure of synchronization between cells in a circuit, computed from phase correlation as


$$C_{x\,y}\,\left(\tau \right)=\left\{ \begin{array}{c} \frac{1}{N-\tau }\,\sum_{n=1}^{N-\tau }x_{n+\tau }\,Y_{n},\tau \geq 0\\ C_{x\,y}\,\left(-\tau \right),\tau <0 \end{array}\right.$$

where x_*n*_, y_*n*_ are normalized signals of length N, and SI ranges [0;1].

Furthermore, we summarized the information content of Ca^2+^ activity dynamics by computing Markovian entropy. Briefly, this method characterizes the level of predictability of transition between defined states of a system. For the purposes of our analysis, we defined states based on activity level, and we discretized single-cell Ca^2+^ activity trains into an alphabet of 2 (corresponding to 2 states states). We then used the discretized data to generate a transition probability matrix, from which we computed single-cell information entropy using Shannon’s formula:


$$E=-\sum_{i=1}^{n}p_{i}\,l\,o\,g_{2}\,\left(p_{i}\right)$$

where p_*i*_ is the value of one entry in column i, and n is the number of states.

This leads to a single-cell Markov entropy value ranging between 0 and 1, which stands as an aggregate measure of predictability of state transitions for an individual neuron: a high entropy value close to 1 suggests a neuron with less predictable dynamics, whereas a low entropy close to 0 points to a neuron with more predictable dynamics.

Finally, binary, undirected functional connectivity (FC) was computed for each condition (Figure 3D) using a previously described pairwise phase correlation method (Patel et al., 2015). Three metrics were chosen to quantify network function: functional connectivity density as a marker of overall connectivity, average characteristic path, and clustering coefficient as measures of network integration and segregation, respectively. Characteristic path and clustering coefficient for each network computed using the Brain Connectivity Toolbox (Rubinov and Sporns, 2010), are reported as scaled values after referencing to the average values obtained from 1,000 random control networks of the same size and connection density.


$$F\,C\,d\,e\,n\,s\,i\,t\,y=\frac{\sum_{N}\left(\sum_{j}c_{i\,j}\right)}{N\times \left(N-1\right)÷2}$$

where c_*ij*_ is pair-wise binary connectivity value, N is total number of nodes in the network;


$$c\,h\,a\,r\,a\,c\,t\,e\,r\,i\,s\,t\,i\,c\,p\,a\,t\,h\,l\,e\,n\,g\,t\,h=\frac{\sum_{i\neq j}n_{i\,j}}{N\times \left(N-1\right)}$$

where n_*ij*_ is pair-wise path length value:


$$c\,l\,u\,s\,t\,e\,r\,i\,n\,g\,c\,o\,e\,f\,f\,i\,c\,i\,e\,n\,t=\frac{1}{N}\,\sum_{i=1}^{N}C_{i}=\sum_{i}\frac{2\,t_{i}}{k_{i}\times \left(k_{i}-1\right)}$$

where C_*i*_ is the clustering coefficient of node i.

### Statistical analysis

Statistical analysis was performed with JMP statistical analysis software. Comparisons between fully coated dissociated cultures and neuronal islands were performed with a Welch’s test. Differences in island population activity and network metrics described above (active population, event rate, SI, FC density, path length, and clustering coefficient) were assessed with one-way ANOVA testing with Tukey’s *post hoc* analysis. The added effect of culture maturation over time or drug treatments was assessed with two-way ANOVA testing with an interaction term (group *drug/time point). Normality was assessed and non-parametric tests were performed as necessary. Our different group sizes were influenced by culture survivability, which favored low-density cultures. To examine the potential complication of sample group size bias, we randomly selected the same number of samples from the larger, low-density group (*n* = 6) that were used in the medium and large density category. We repeated this random selection of a data subset within the low density group 10 times, and we did not observe a significant change in the trends observed in activation rate, synchrony and activity rate.

## Results

*Neuronal islands are more densely populated and active than the traditional dissociated cultures.*

We assessed the viability of the neuronal islands platform over time periods and conditions used for our regular neuronal culture (traditionally plated dissociated cultures). Representative examples of immunofluorescent staining of three island sizes ranging from 750 to 1,300 um show selective co-localization of cellular nuclei with the patterned laminin, connected by an extensive network of nuclear processes emerging as early as 10 DIV (Supplementary Figure 2).

We further probed the functional state of the island circuits (*n* = 32) by monitoring their Ca^2+^ activity at DIV 10 and comparing it to that of traditionally plated dissociated cultures (*n* = 10). The analysis pipeline as described in the Section “Materials and methods” (Supplementary Figure 3) yielded segmentation masks with a significantly smaller average number of cells in island cultures than in the traditionally plated cultures (Supplementary Figures 3A–C, 180 ± 12 (islands) and 390 ± 56 cells (traditional), *p* < 0.001), as expected given the smaller surface area covered. However, increased attachment and survival of neurons onto the patterned islands was revealed by the significantly larger cellular density when compared to traditionally plated controls [Supplementary Figure 3D, 178 ± 15 (islands) and 103 ± 15 cells/mm^2^ (traditional), *p* < 0.01]. Furthermore, activity rate was correspondingly increased in islands (Supplementary Figure 3E), 9.1 ± 1.3 (islands) and 3.9 ± 2.3 events/min (traditional), *p* < 0.01), but the pattern of activity as described by synchronization index was independent of the culture platform (Supplementary Figure 3F, 0.55 ± 0.02 (islands) and 0.53 ± 0.04 (traditional), *p* > 0.05).

*Island size does not influence activity and network parameters.* The first design parameter that we investigated, island size, was fixed at three levels to assess whether new activity features emerge as the spatial extent of a network is increased. Island diameter was varied from 750 to 1,300 um, corresponding to surface areas between 1.1 and 2.2 mm^2^ (small: 1.11 ± 0.22 mm^2^, *n* = 4; medium: 1.45 ± 0.15 mm^2^, *n* = 4; large: 2.23 ± 0.18 mm^2^, *n* = 3), and we focused our attention on the low cellular density level from before (<175 cells/mm^2^) as a means to ensure good spontaneous activity.

Representative examples of Ca^2+^ activity in islands of three sizes at 10 DIV revealed rich activity patterns across all island sizes tested (Figure 3). Comparison of both activity and network metrics at 10 DIV confirmed that island size did not have a significant influence on any of the metrics (Figure 3D, left panels). To further confirm that activity patterns observed are reproducible, we repeated activity monitoring at 14 DIV, when neuronal islands were expected to have reached full maturation (Chiappalone et al., 2006). By 14 DIV, the fraction of an island’s population engaged in spontaneous activity decreased significantly (Figure 3D, *p* < 0.01), while the activity rate of the cells still integrated in the circuit increased (Figure 3E, *p* < 0.05). The level of synchronization and all network metrics were independent of island size or developmental time point (Figures 3F–I). Therefore, we conclude that island size, within the tested range, is not a significant driver of circuit activity.

*Increasing cellular density biases neuronal islands towards a less active, but more reproducible, state of activity.*

We then investigated the role of cellular density in shaping the activity and network metrics of neuronal islands. Visual inspection of representative raster plots of increasing cellular density (Supplementary Figure 4) revealed an overall decrease in activity level and the appearance of sparse synchronous events followed by long periods of activity silence.

**FIGURE 4:**
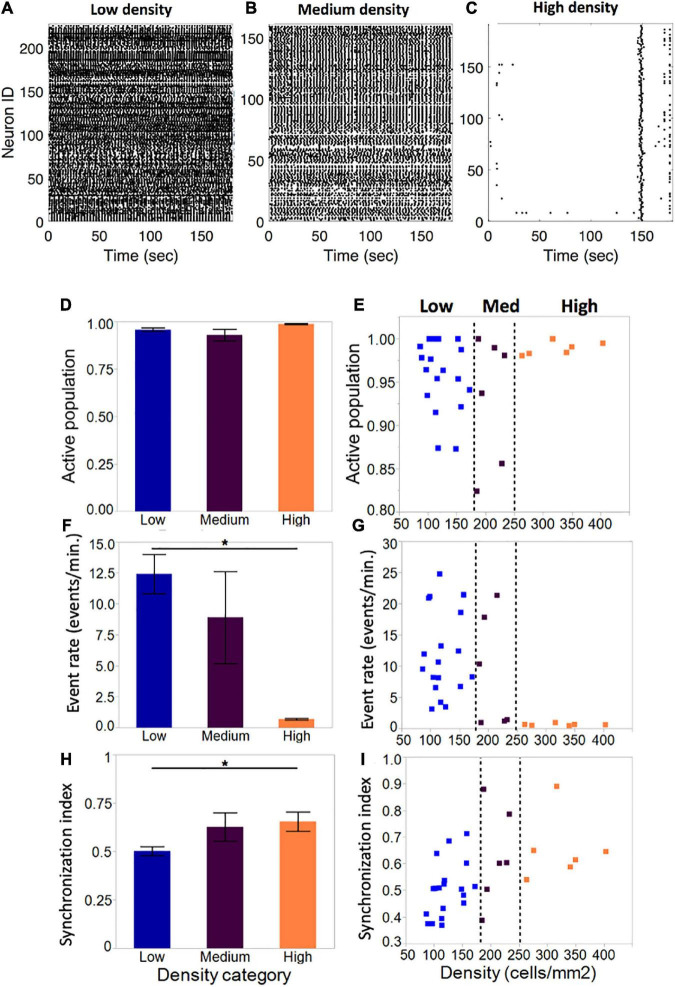
Activity metrics in neuronal islands of varying cellular densities **(A–C)**. By DIV 10, the vast majority of neurons in islands were functionally integrated in spontaneous Ca^2+^events, independent of the cellular density of the culture (low: *n* = 19; medium: *n* = 6; high: *n* = 6). **(F,G)** Event rate, defined as the average number of distinct calcium transients in the island per neuron per minute, decreased significantly as cellular density increased. Averages for each category were computed as a mean of the single-cell event rates of all of the cells within a given category. **(D,E)** Activity became increasingly coordinated as cellular density was increased. **(H,I)** The distribution of island samples was more variable in low-density and medium-density islands for both the fraction of population that was active, as well as the average island event rate, with an apparent threshold of cellular density (250 cells/mm^2^) which biased islands to a state of low-rate, highly synchronous activity. No threshold effect was observed in the distribution of synchronization index values. Significance level for statistical testing was set at 0.05 (*p < 0.05).

To further describe which aspects of neuronal activity are potentially modulated by cellular density, we focused on three specific metrics: fraction of population active, activity rate and synchronization index in three cellular density island groups (low: < 175 cells/mm^2^, *n* = 19; medium: 175–250 cells/mm^2^, *n* = 6; high: >250 cells/mm^2^, *n* = 6). We chose these 3 metrics for our analyses as they are robust yet sensitive to changes that appear after injury. Of note, the range of densities was limited on the low end by the minimum connection density required for cultures to sustain spontaneous activity, and on the high end by the maximum density which would not lead to the formation of large, unhealthy neuronal aggregates. In general, activity ranged from more active (low, medium density) to periodic, bursting activity (high density) (Figures 4A–C).

The average fraction of the island population that was spontaneously active did not vary significantly across density categories (Figure 4D, low: 0.96 ± 0.009, medium: 0.93 ± 0.03, high: 0.99 ± 0.003). Closer investigation of the distribution of active population fractions across all of the islands (Figure 4E) confirmed close to full participation of cells to spontaneous island activity (>85% of neurons active).

Despite equal fractions of active cells, event rate decreased significantly as island density increased (Figure 4F). Low-density islands displayed high activity rates (12.4 ± 1.6 events/min), which decreased in medium-density islands (8.9 ± 3.7 events/min) and settled into low activity rates in high-density islands (0.69 ± 0.07 events/min). Of note, the variability of activity rates observed also decreased from highly variable at low density, to highly consistent at high density. Furthermore, the distribution of individual island activity rate (Figure 4G) revealed two clear regimes of activity: highly variable high activity at low cellular density, and highly consistent low activity at high cellular density, separated by a transition zone in which medium cellular density could bias an island towards either of the two activity regimes.

The average synchronization index, a coarse descriptor of activity pattern, increased with increasing cellular density (low: 0.50 ± 0.02; medium: 0.61 ± 0.75; high: 0.65 ± 0.82), suggesting that activity among neurons within an island is more correlated in denser islands (Figure 4H). The distribution of values showed a trend for increased synchronization with cellular density, but not a clear threshold of transition between island groups as in the case of activity rate (Figure 4I).

**FIGURE 5:**
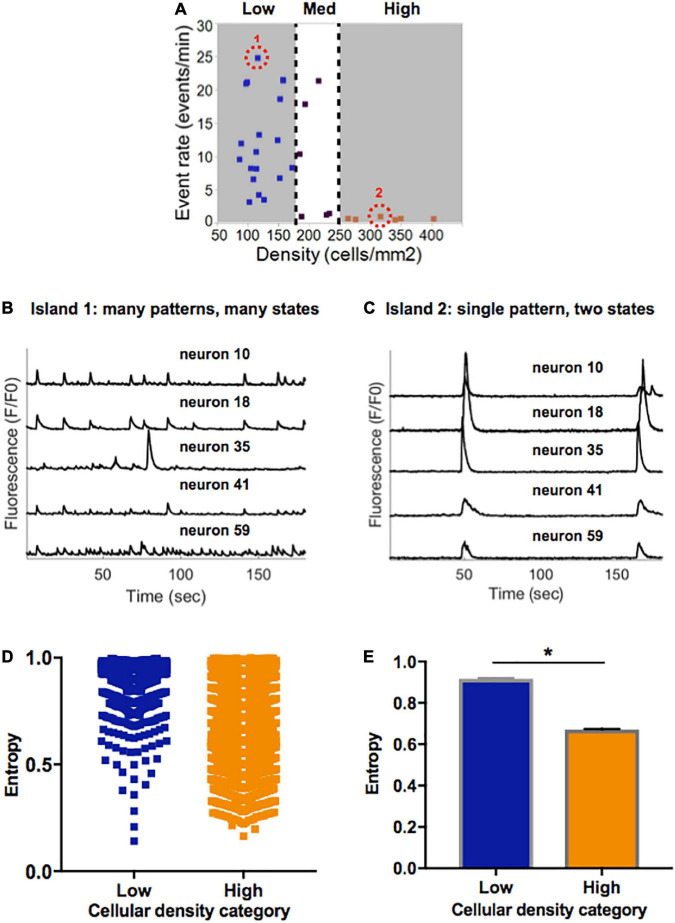
Information entropy in islands of varying densities. **(A)** Selected representative islands form the low- and high-density category. **(B)** Five example traces of individual neurons within Island 1 (low density) display a rich pattern of activity. **(C)** Five example traces of individual neurons within Island 2 (high density) only achieve one pattern of activity which is highly synchronized. **(D)** Distribution of single cell entropy values at baseline across neurons in low (*n* = 1471) and high (*n* = 3024) density circuits. Each point represents an individual neuron within an island of the designated density. **(E)** Aggregate average entropy levels are significantly higher in low compared to high cellular density islands (0.92 ± 0.002 vs. 0.67 ± 0.003, *p* < 0.0001). Significance level for statistical testing was set at 0.01 (*p < 0.01) in Mann–Whitney test.

Further inspection of the single cell patterns within selected islands of various densities revealed more subtle differences not only in the average activity, but also in the patterns of activity that the island circuit can achieve. Qualitative assessment of single-cell traces from representative examples of low and high density islands (Figure 5A) revealed that neurons in a low density circuit can support many patterns of activity (Figure 5B), and therefore function in many activity states. By contrast, a high density circuit enables a single pattern of synchronous activity in all neurons (Figure 5C), suggestive of the circuit and individual neurons functioning in only two states: on and off.

To better characterize the propensity of an island to function in a pattern of activity that is more spontaneous versus limited to only two states, we implemented a single-cell Markovian entropy metric as described in Section “Materials and methods.” This allowed us to compare the distribution of entropy values for the activity trains of individual neurons in low density compared to high density circuits. For clarity of analysis, we focused our computations on the low- and high-density circuits only, as those had the most distinct activity features. Qualitative analysis of the distribution of entropy values across all neurons in each cell density category raveled a tendency for higher entropy values in neurons that were part of a low-density circuit (Figure 5D). When computed across all neurons in each island density category (Figure 5E), average entropy was significantly higher in low-density than high-density circuits (0.92 ± 0.002 vs. 0.67 ± 0.003, Mann–Whitney *p* < 0.0001). As defined, a higher single-cell entropy means a less predictable transition between states of activity, or a system that can more easily create spontaneous patterns of activity (i.e., the rich activity patterns in low density islands). A lower entropy level marks a system whose state transitions, here representing activity patterns, are more predictable (i.e., regular synchronous bursts seen in high density islands).

Taken together, these results point to spontaneous activity in small neuronal islands being driven by cellular density. We define “flexibility” as the ability of a system to achieve a diversity of states and functions given a common structure–e.g., the ability of islands of a given density to give rise to multiple activity patterns. We thus conclude that increasing cellular density reduces the flexibility of a circuit and biases it towards a narrow range of low-rate, synchronous and highly predictable activity. In contrast, less dense islands are more flexible circuits that are capable of achieving a broad range of rates and patterns of activity.

*Cellular density correlates with functional networks of increased density and low segregation*.

We employed several network analyses tools to evaluate functional properties of the different island circuits. Based on functional connectivity maps derived from Ca^2+^ activity (see Section “Data analysis”), we computed node degree density, characteristic path length and clustering coefficient to evaluate basic network properties.

**FIGURE 6:**
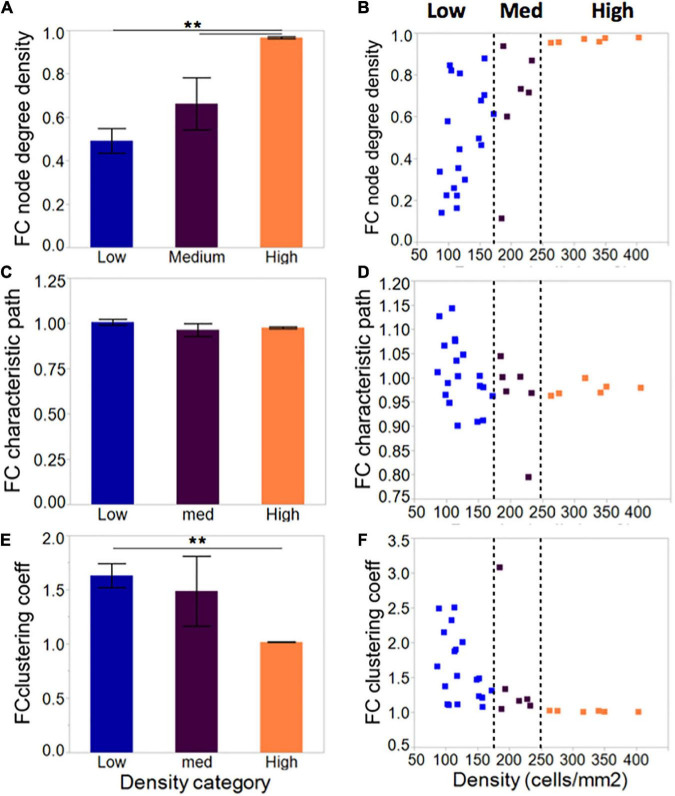
Network metrics in neuronal islands of varying cellular densities. **(A)** Phase correlation-based functional connectivity was increased in islands of higher cellular density, pointing to more densely connected neurons within the high-density islands. All values were scaled to the total number of connections possible given a network’s size, such that all difference observed reflect increased connectivity rather than the increased availability of neighbors to connect to. This difference was accompanied by a corresponding reduction in clustering coefficient **(E)**, a measure of network segregation, but no significant difference in average characteristic path **(C)**, a measure of network integration. Both characteristic path and clustering coefficient were referenced to average values derived from size- and degree- matched random control networks for each island. Therefore, a value of 1 for characteristic path and clustering coefficient indicates that the network is very similar to a random one. **(B,D,F)** As for activity markers in Figure 4, a cellular density threshold at 250 cells/mm^2^ marked the transition between networks with highly variable characteristics (low cellular density islands) to networks with very well-defined properties (high cellular density islands). Significance level: **p < 0.01.

Compared to low cellular density networks, islands rich in neurons formed more densely connected networks when taking into account the number of neurons available and the total possible connections among them (Figures 6A, C, E–low: 0.49 ± 0.06; medium: 0.66 ± 0.12; high: 0.97 ± 0.001). This did not influence the characteristic path length, which averaged close to 1 for all island groups. Clustering coefficient, a measure of network segregation into smaller units, was inversely correlated with cellular density: values of 1 computed for high-density islands are indicative of networks that are as segregated as a random network; values of 1.5 observed for low-density islands point to networks that include more clusters than random, thus are more segregated than their matched controls. Based on the combination of characteristic path and clustering coefficient values, our island cultures fall in the category of random networks.

As with activity metrics, inspection of the distribution of network metrics across groups (Figures 6B, D, F) revealed two regimes of network properties separated by a cellular density threshold of 250 cells/mm^2^: highly variable networks ranging from very sparse to full connectivity at the low cellular density level; and a robust, highly connected set of networks at high levels of cellular density.

Overall, network results agree with activity results, wherein low-density islands can achieve networks with a range of properties, while high-density networks are primed into a single state of high functional connectivity.

*Low-density circuits can be functionally converted to a high-density activity state with pharmacological removal of synaptic inhibition.*

**FIGURE 7:**
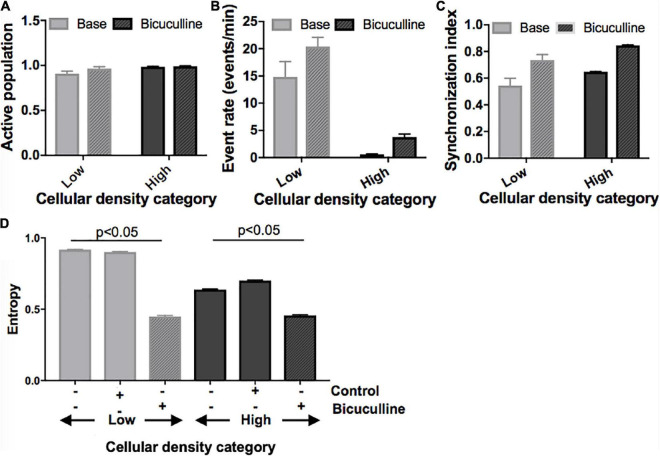
Activity response to alterations in excitatory/inhibitory balance in neuronal islands of different cellular densities. **(A–C)** Solid fill bars. Summary activity statistics of islands reveal that, s before, event rate at baseline was dependent on cellular density: low density islands showed significantly higher rates of activity than high density islands (two-way ANOVA *p* < 0.05 for “cellular density” factor). Synchronization index also showed a trend of higher synchronization at higher island density, but was not significant, likely due to the small sample size. **(A–C)** Patterned fill bars. Removal of inhibitory synaptic transmission after treatment with Bicuculline, a GABA_A_ inhibitor (*n* = 11), recruited the remaining non-active population of neurons, and significantly increased island activity rate and synchronization level (two-way ANOVA *p* < 0.01 for “drug” factor). This effect was uniform across islands density groups (“density” × “drug” interaction term not significant). **(D)** The addition of Bicuculline also significantly decreased entropy compared to baseline values and to the control (adding inert MEM). This effect was seen in both low- and high-density islands (two-way ANOVA *p* < 0.05 for “drug” factor).

Next, we divided islands to probe whether differences in activity seen between islands of different densities can be attenuated by biasing the E/I ratio. Removal of inhibitory transmission did not recruit a significant number of additional neurons to the active population in any of the island groups (Figure 7A) but increased the activity rate and synchronization index (Figure 7B; activity rate: *p* < 0.05; synchronization index: *p* < 0.01). This effect was uniform across islands of different cellular densities, and existing relative differences between groups were maintained. Specifically, low- and high-density islands both showed significant increases in activity after drug treatment compared to their baseline values (low: 14.8 ± 2.8 vs. 20.4 ± 1.7 events/min; high: 0.62 ± 0.05 vs. 3.78 ± 0.56; *p* < 0.05), but remained significantly different from each other post-drug (low vs. high, *p* < 0.05).

At the network level, removal of inhibition increased the node degree density without altering the relative relationship between groups (data not shown): low density islands were significantly less functionally connected than their high-density counterparts, both initially as well as after Bicuculline addition (low: 0.57 ± 0.13; high: 0.97 ± 0.01, *p* < 0.05; Figure 7C). Characteristic path and clustering coefficient computed relative to control random networks were not altered by the change in E/I balance at any cellular density level.

Lastly, we compared changes in entropy (defined as described in Section “Materials and methods) between cultures treated with Bicuculline and controls (Figure 7D). As before, baseline entropy level was significantly higher in cultures of low density (0.92 ± 0.003 vs. 0.64 ± 0.004, *p* < 0.05). The reduction of inhibitory tone with Bicuculline (and corresponding relative increase of E/I), but not the addition of control media, lead to a significant decrease in entropy in low-density islands, similar to values measured in high density islands (0.45 ± 0.006 vs. 0.46 ± 0.005).

Taken together, these results point to the E/I ratio as a key mechanism mediating the observed cellular density-induced differences in activity. We propose that cellular structure guides E/I balance, which in turn determines the flexibility of activity and network state in a circuit. Specifically, we hypothesize that low-density islands start with high inhibitory tone (low E/I ratio), which allows them to achieve a large range of activity rates, patterns, and network structures with higher entropy values. They are therefore more flexible. Oppositely, high-density islands have a high E/I ratio, which biases their activity and functional network structures into a single state of very predictable high activity, as there is little negative feedback to control the frequent, synchronous activity bursts. This translates into lower baseline entropy values and lower flexibility. Pharmacological increase of the E/I ratio with Bicuculline appeared to convert low-density islands to less flexible (lower entropy) circuits similar to the high-density islands, suggesting that flexibility is a functional characteristic of a circuit that is dependent on the E/I balance, rather than a direct consequence of structural properties such as cellular density.

*Cellular density does not influence final susceptibility to cumulative injury of island nodes, but it alters dynamic response to gradual injury.*

Given that activity was the main differentiator between islands of different cellular density and based on prior studies showing the importance of baseline activity on response to injury, we hypothesized that low and high density islands would respond differently to injury. To test this hypothesis, we developed a mechanical injury model which inactivates single cells (see Section “Microablation injury”) to probe whether the severity of injury required to completely silence activity, or the rate at which activity is lost in response to injury, measured as fraction of cells still active, is a function of cellular density. For clarity of results, for this analysis we focused on small and large islands only, where we had observed the most significant activity differences.

**FIGURE 8:**
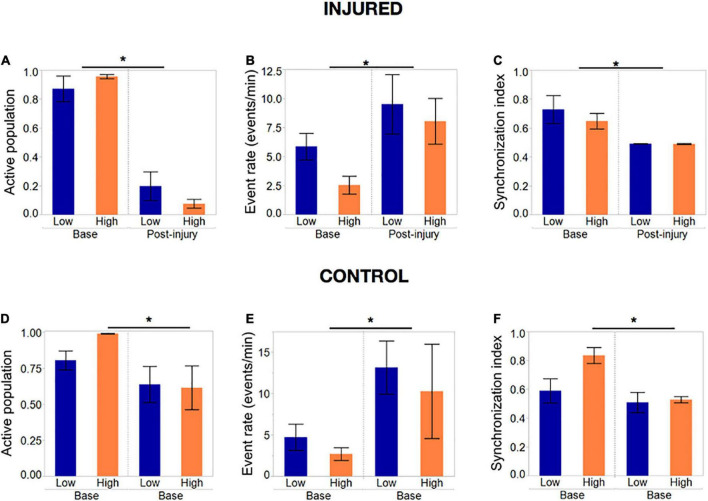
Activity metrics in response to complete silencing of neuronal islands’ population. **(A–C)** Activity metrics at the end of the injury protocol; sets of five neurons were inactivated with the tap injury model until there no activity was detected by visual inspection. The active population was reduced significantly (but not 0) in both island groups, with neurons that remained active showing significantly higher rates of activity and a decreased synchronization index. Low- and high- density islands were not significantly different from each other post-injury. **(D–F)** Activity metrics in control dishes that underwent the same illumination protocol, but no tap injury, revealed a significant decrease in the fraction of the population active, coupled with an increase in the activity rate and decrease in synchronization. Significance level was set at *p < 0.05.

Summary statistics comparing the baseline and final post-injury recording of each island confirmed that most of the active population had been silenced in both island groups (post-injury active population fraction 0.2 and 0.04, *p* < 0.01) by the end the injury protocol (Figure 8A). The remaining active neuronal population had significantly increased activity rates (Figure 8B) compared to the baseline recording (9.1 and 7.9 events/min compared to 5.6 and 2.5 events/min, *p* < 0.05) and decreased synchronization levels (Figure 8C).

To our surprise, control islands which were exposed to the same illumination protocol but not the mechanical injury, also suffered a loss of active neurons, albeit not as pronounced as in the injury dishes (Figure 8D). This was correlated with an increase in activity rate and decrease in synchronization level to an extent that was comparable to the injured dishes (Figures 8E, F). We attribute this finding to possible photo toxicity from the long exposure to laser and bright field light required to perform the sequential injuries. However, the loss of active neurons was significantly more pronounced in the injured group, therefore suggesting that only a small part of the response in injured islands is due to photo toxicity, while the bulk of loss in activity is due to injury.

Given that the fraction of active neurons was most significantly affected by injury, we decided to use this metric as a means of assessing island resilience. Several qualitative trends emerged: the loss of activity was not linearly correlated to injury, but rather complete silencing occurred once a threshold of injury was attained; many of the island in both cellular density groups lost activity at very low levels of injury; overall, low-density islands (gray) maintained a higher active population fraction up to higher levels of injury than their high-density counterparts (black).

**FIGURE 9:**
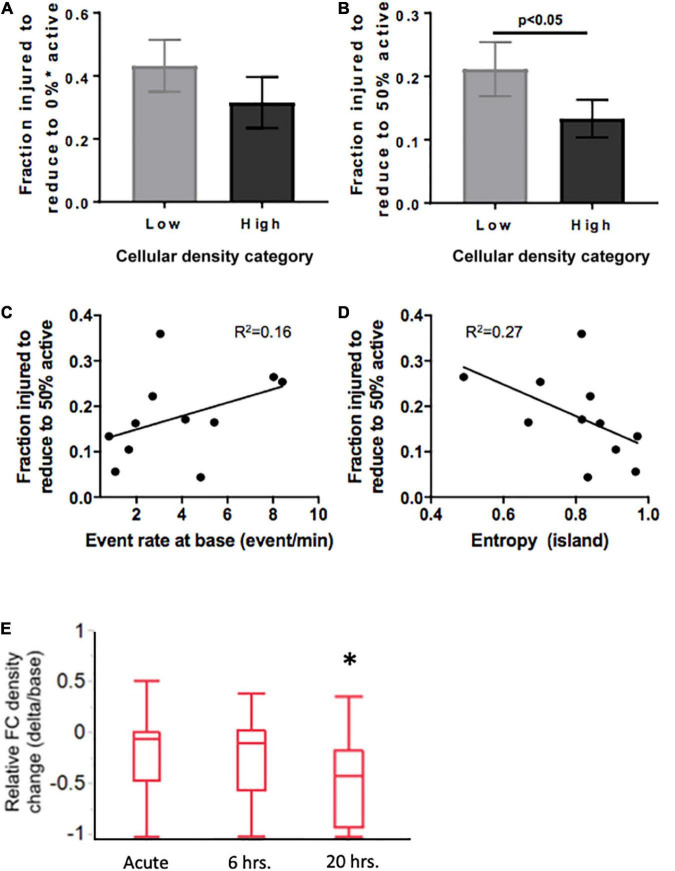
Mediators of resilience in response to sequential microablation of neuronal islands’ population. **(A)** The total fraction of cells that need to be eliminated from an island to completely reduce their active population to 0 was not dependent on cellular density. **(B)** High density islands required significantly lower injury levels to reach the half-point of activity level (50% of population maintains activity). This response was only moderately dependent on the baseline activity of the island as described by baseline event rate **(C)** or entropy **(D)**. Inactivating 15–25% of neurons in high density islands led to no significant change in relative functional connectivity density either immediately or 6 h after injury, but did lead to a significant reduction in the relative functional connectivity density 20 h after microtrauma **(E)**. Significance level was set at *p < 0.05.

Summary statistics of the injury level required to collapse activity (Figure 9A) confirmed that while the average injury level required to silence low-density islands was higher than for high-density islands [0.31 (low density) vs. 0.42 (high density) fraction of cells injured], the difference was not statistically significant (Figure 9B). A closer look at the profile of activity throughout injury revealed that the rate of decay of activity with injury varied among islands. We therefore computed the level of injury required to reduce the active population to less that 50% of the baseline value, to account for differences in the rate of activity collapse in the two islands groups. High-density islands lost 50% of their active population at lower injury levels than low-density islands (Figure 9C–0.12 vs. 0.22 fraction injured, *p* < 0.05).

Knowing that cellular density functionally guides activity, we were interested in assessing if the resilience to injury could be predicted by any of any of the specific activity properties of the circuit. Specifically, we tested whether either baseline activity rate or entropy of a circuit would predict its resilience to injury defined as the fraction of neurons injured to silence 50% of the initial active population. Only modest correlations were observed between event rate or entropy of islands regardless of cellular density group (Figures 9D, E, *R*^2^ = 0.16 and 0.27, respectively). Therefore, island activity was overall highly susceptible to the disconnection of individual nodes, a response which was largely independent of initial activity.

Finally, after establishing thresholds for activity reduction in both low and high density circuits, we next examined the longterm changes in connectivity after trauma. Focusing on high density circuits, we observed that injury to 15–25% of the neurons within a circuit did not significantly change the relative functional connectivity density either immediately after injury or 6 h after injury (Figure 9E). However, the relative functional connectivity density significantly decreased 20 h after injury, indicating that microtrauma to the network can lead to eventual loss in functional connections across the circuit.

## Discussion

In this study, we characterized the function of small neuronal circuits (islands) and their response to pharmacological and mechanical perturbations. We focused our attention on two structural design parameters–size and cellular density of island–and assessed their influence on neuronal circuit function. Key findings include that cellular density, but not size, dictates the range of states of activity that a circuit can achieve spontaneously–a property we refer to as “flexibility”- and that flexibility can be functionally altered by biasing the excitatory/inhibitory balance. We then tested if flexibility of a circuit affected the resilience of a circuit to sequential inactivation of nodes in the circuit, finding that resilience to injury is positively correlated with flexibility: circuits that can achieve more states of activity are more resilient to injury. We interpret these results to emphasize the role of structure in guiding function in the form of activity, both spontaneously and in response to injury.

The structure-function relationship has been of interest to the neuroscience community as an important mediator of physiological and pathological conditions of the nervous system (Honey et al., 2009; Caeyenberghs et al., 2013; Sporns, 2013; Ocker et al., 2017). *In vitro* early work attempting to control function by restricting neurons to pre-defined patterns established one simple structural feature, cellular density, as a key driver of electrical activity levels in hippocampal neurons (Chang et al., 2001). More recently, Boehler, Leondopulos (Boehler et al., 2012) found that higher levels of pattern complexity, leading to increasing density of connections, biases activity towards low-rate, long-duration network bursts in hippocampal networks. Pan et al. (2015) further expanded the study of structure-function relation to the meso-scale by assessing the impact of connectivity strength on the propagation of activity between small neuronal clusters. Some past studies showed that, over time after plating the *in vitro* culture, these different states can emerge and switch the network from sporadic activity into a more coherent bursting state (Wagenaar et al., 2006; Sun et al., 2010). Our observations show that we can titrate both island size and cellular density to toggle between these two general states observed in separate past studies–lower density neuronal networks to achieve an active network with continual activity patterns throughout the network, while higher density networks shifted the network into an episodic bursting state. We extend these observations in examining how these multi-state networks are altered after a mechanical perturbation which gradually inactivated nodes within the microcircuit. Our overall goal was to understand if features of the microcircuit would influence how traumatic injury affects the performance of the circuit.

Our measures of circuit function used many metrics from past studies as these have been shown to be sensitive metrics that have major implications for both healthy and pathological conditions. Under physiological conditions, variations in activity rate and synchronization are means to ensure diversity of function across micro- and macro- circuits in the brain (Buzsaki and Draguhn, 2004). The diversity of neural patterns is thought to emerge from intrinsic properties of neurons as well as the strength of coupling between them (Penn et al., 2016). Loss of activity and synchronization within a small microcircuit likely leads to an alteration in the synchronization of larger areas connected to this region, and many large-scale imaging studies have shown long-lasting alterations of activity of the functional and structural brain networks after TBI (Eierud et al., 2014; Cantu et al., 2015). Our results of both activity rate and synchronization at the microcircuit level being strongly influenced by cellular density establishes the importance of structural properties in guiding function, starting at levels of architecture as low as the microscopic mesoscale. These observations also imply lower density or less mature cultures–both of which reduce the likelihood of bursting–may provide more resilient to microtrauma.

We expanded the scope of simple activity metrics by computing entropy as an aggregate measure of the information conveyed by the activity patterns within a microcircuit. We found that high-density microcircuits displaying periodic and simultaneous activation of the entire network carry very little information content (low information entropy), as the circuit exists in primarily two states–active or inactive. In comparison, low-density circuits showed a large diversity of patterns among the neurons and, as such, the information contained in the pattern was much higher than a binary circuit (higher information entropy).

These findings confirm and expand on prior investigations of cellular density and its impact on activity, which showed that areas of high neuronal density initiate propagating population bursting activity (Feinerman et al., 2007; Brewer et al., 2009). Mechanistically, this could be a way to allow different sub-circuits of the brain to perform different functions and convey information content based on their activity patterns: a low-density circuit provides fluctuating amounts of information which can be easily adjusted; while a high-density circuit functions as a reliable relay to either convey or gate information. Our findings suggest that such division of function occurs at levels of architecture as low as microcircuits of a few hundred neurons.

We further expanded on past studies of injury in dissociated microcircuits by considering descriptors of the network structure–network density, characteristic path length and clustering coefficient–as possible key variables that could define the functionality of our neuronal islands and the subsequent response to graded trauma. Graph theory metrics offer several key prospective advantages for understanding neural network function. Markers of network efficiency, segregation and integration of signaling are robust (Deuker et al., 2009), scalable (van Wijk et al., 2010) and sensitive to changes that occur in neuronal circuits over development (Woiterski et al., 2013; Baker et al., 2015; Hutchison and Morton, 2015) and in disease (Dubbelink et al., 2014; Wu et al., 2015). Specifically, past work has shown that TBI causes macroscale changes such as disruption of the default mode network, reduction in efficiency and loss of modular structure (Pandit et al., 2013; Caeyenberghs et al., 2014; Han et al., 2014; Fagerholm et al., 2015), as well as microscale disruption in network modules and small-world properties (Srinivas et al., 2007; Patel et al., 2012, 2014). Our results of increased network parameter variability in islands of low density point to the critical role of structural parameters in guiding the functional network outcome, and to the emergence of this feature at the microscopic network level.

**FIGURE 10:**
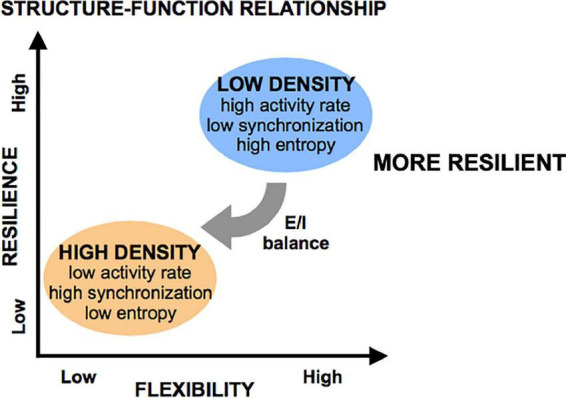
Flexibility and resilience as a function of structural features. Low-density islands are inherently more flexible (defined as having higher entropy) than high density islands, a property which is mediated through the excitatory/inhibitory (E/I) balance (low in low-density islands, high in high-density islands). When subjected to injury, low-density islands are more resilient to low levels of injury than high-density islands. However, it is possible to significant degrade both circuit types if the microtrauma is severe.

Given these activity and network metrics, we defined the concept of “flexibility” as the ability of a system to achieve a diversity of states and functions given a set structure. More diverse patterns of activity that can convey more information in less rigid functional networks were considered more flexible because they can give rise to a host of functional states. We conclude that circuits of low cellular density are more flexible than those of high cellular density, as these low-density circuits spanned a large range of activity rates, synchronization index, and high entropy values, coupled with a wide range of functional network properties. Alternatively, increasing cellular density leads to a very reproducible/predictable state of low activity, high synchronization, low entropy, and set network structure of high density, low integration, and low segregation. A schematic of these functional states appears in Figure 10.

Contrary to cellular density, circuit size did not influence any of the activity- or network- based island properties. This is not surprising, given that the small range of our island sizes would not allow for a true distance-dependent connectivity matrix to develop. Indeed, all of the islands in these studies showed a random functional connectivity network, suggesting that the underlying structural network is not very diverse among the different island sizes and similar to findings in hippocampal two-dimensional neuronal networks by Srinivas et al. (2007). It is likely, however, that increasing the size of our islands would eventually produce a distant-dependent structure and novel functional properties such as network clustering and complex meso-scale structure would emerge, as reported in previous network studies of *in vitro* systems (Downes et al., 2012; Malmersj et al., 2013; Santos-Sierra et al., 2014; Schroeter et al., 2015; Nigam et al., 2016). However, our current study was limited to viewing the entire island structure simultaneously and therefore prevented us from studying larger physical sizes where these properties could appear.

The excitatory/inhibitory (E/I) balance is a known modulator of activity and synchronization in neuronal systems (Maeda et al., 1995; Brunel, 2000), therefore we hypothesized that the E/I balance would be a potential mechanism that could convert a flexible network into a much less flexible network, taking advantage of the shift in flexibility we observed across cellular density. This model would be consistent with reports in post-TBI epilepsy literature, where increased cellular density (from aberrant neurogenesis triggered by injury) leads to increased activity, and coupled with injury-induced dysregulations in the E/I equilibrium, primes the circuit for an inflexible, pathological state of synchronous activity (Golarai et al., 2001). Our finding of reduced flexibility following the reduction of inhibitory synaptic transmission with Bicuculline (a GABA antagonist, effectively increasing the E/I ratio) in otherwise flexible low-density islands confirms that the E/I balance is a functional modulator of circuit flexibility. It is intriguing to consider the broader significance of the E/I balance shifting flexibility of the circuit, as several reports show that the hippocampus shifts its E/I balance after TBI and becomes a more synchronized (i.e., less flexible) circuit that is conducive to post-traumatic epilepsy (Ding et al., 2011; Cantu et al., 2015; Nichols et al., 2015; Villasana et al., 2015). The opposite manipulation, enhancement of the inhibitory transmission with Muscimol (GABA agonist), completely silenced activity in all circuits, and was thus less informative about the effect of E/I variations on circuit flexibility.

With an assessment of the flexibility that was created with islands of different density, we tested directly how flexibility would influence the degradation of spontaneous activity with mechanical trauma. Several studies have established that activity rate, synchronization, and network integration, both at the single-cell and network level, are important direct and indirect predictors of a circuit’s response to injury (Geddes-Klein et al., 2006; Patel et al., 2014). In general, high activity levels within individual neurons tend to protect the neuron from mechanical damage, although there is evidence that activity differences across distinct brain regions may not follow the same principle, likely from a different distribution of mechanically sensitive receptors across the two regions (Kang et al., 2015). Our first finding that neuronal islands of both densities showed gradual reduction in the number of active neurons with increasing injury levels, until a threshold was reached and activity collapsed, is in agreement with a similar computational model of targeted deletion of nodes in a network, showing resilience to random deletion up to removal of 50–60% of the nodes in the network (Rubinov et al., 2009). The difference in the threshold value between the computational model and our *in vitro* circuits could be due to additional physiology and pathology that are not captured by the computational model, such as triggering of secondary injury cascades. Importantly, the fraction of neurons required to completely silences activity, was not different between island groups, suggesting that when subjected to enough loss of nodes and the afferent connectivity, circuits ultimately lose the ability to spontaneously convey information. This observation is consistent with the concept of percolation in neuronal systems described by Breskin et al. (2006) and more recently by Breskin et al. (2010) and Eckmann et al. (2010), wherein the probability of a neuron to fire is determined by its connectivity; thus, removing a large enough number of neurons from the circuit will ultimately bring it below the threshold for activity maintenance. This is particularly relevant since the threshold for percolation is dependent on the network size (Tlusty and Eckmann, 2009), therefore in small networks, such as our neuronal islands, the number of neurons still integrated in the circuit can quickly fall below the threshold.

A more intriguing trend appeared when examining the ability of a circuit to preserve at least 50% of the initial activity with increasing levels of injury, another indicator of its resilience, revealed that low-density islands can maintain activity in half of their population up to higher levels of injury. This suggests that they have increased partial resilience to injury, and this effect is at least partially correlated to activity rate, which is generally higher in low-density islands. We propose that the increase in partial resilience in low-density islands is a direct effect of their increased flexibility and higher activity rates, which allow for adaptation to alternative states of activity in the face of injury. By contrast, high-density networks, which have low flexibility and can only function in one state of synchronous low-rate activity, likely starting in localized initiation zones that compete in driving network activity (Feinerman et al., 2007), have no alternative activity pathways, and are likely to collapse as soon as the initiation zone has been inactivated. While in this study we focused our attention to the initial and final states of activity to derive general principles of how resilience and flexibility are related, the ability to monitor circuits through the gradual removal of neurons, coupled with analysis tools to track network dynamics over time (e.g., hypergraphs), will allow us to explore in more detail the transitions through states of activity during injury in future studies. A key unknown is how the innate plasticity mechanisms in these cultures these transition states. Across several dozen cultured islands, we attempted to induce plasticity by treating the cultures with bicuculline, a method used in past dissociated cultures to alter synaptic strength (Arnold et al., 2005), However, we could not detect a consistent change in the dynamics of these uninjured cultures–treatment in some cultures led to a persistence of coordinated activity once the bicuculline was removed, consistent with past work. The synchronization of other cultures, however, were not different from the synchronization prior to treatment. Due to this variability, we could not reliably (and consistently) induce plasticity across all cultures and answer whether plasticity was a key factor in rebuilding injured microcircuits.

In summary, we have developed a robust experimental platform to closely investigate the activity development and response of small neuronal circuits *in vitro*. We non-invasively monitored activity and the resulting functional connectivity networks in small neuronal islands of varying structural properties and probed their response to network-wide pharmacological and mechanical manipulations. Our first finding is that cellular density, a structural feature of the circuit, is an important parameter that modulates the functional activity, information transfer and network states, a property we collectively refer to as “flexibility,” likely *via* the excitatory/inhibitory (E/I) balance. We applied this knowledge to an important unresolved problem, traumatic brain injury, through a model of progressive ablation of individual neurons, and found that cellular density also guides a small circuit’s response to gradual inactivation of its nodes, a property we refer to as “resilience,” likely through modulation of the circuit flexibility.

By establishing that structure, and the resulting function (flexibility), are important factors in guiding the circuit response to injury (resilience), our findings add to our understanding of the structure-function relationship which remains an open question in neuroscience and allow for systematic experimental investigation of the different key parameters that modulate this relationship. Furthermore, they begin a systematic investigation of how known molecular and single-cell mechanisms, such as the E/I balance, lead to the appearance of circuit-wide responses. Future directions to expand on current findings should focus on further understanding the structure–E/I balance –flexibility/resilience relationship by combining in one study manipulations of the E/I balance (as a way to influence circuit flexibility) with inactivation injury to assess resilience.

## Data availability statement

The original contributions presented in this study are included in the article/Supplementary material, further inquiries can be directed to the corresponding author.

## Ethics statement

This animal study was reviewed and approved by the University of Pennsylvania Institutional Animal Care and Use Committee.

## Author contributions

MA and DM conceived and designed the studies, analysis, and wrote the manuscript. MA and OT performed experiments and conducted analysis. All authors contributed to the article and approved the submitted version.
