# Supplementary material for: Flexibility of in vitro cortical circuits influences resilience from microtrauma
Source: Front Cell Neurosci. 2022 Dec 16;16:991740. doi: 10.3389/fncel.2022.991740 (PMC9803265; doi:10.3389/fncel.2022.991740)
Supplement: Supplementary file 1 [file Data_Sheet_1.pdf]

SUPPLEMENTARY MATERIALS

Flexibility of *in vitro* cortical circuits influences resilience from microtrauma

Modupe A. Adegoke[1], **Olivia Teter**[1], **David F. Meaney**[1,2*]

[1]Department of Bioengineering, School of Engineering and Applied Sciences, University of Pennsylvania, Philadelphia, Pennsylvania, United States of America

[2]Department of Neurosurgery, Penn Center for Brain Injury and Repair, Perelman School of Medicine, University of Pennsylvania, Philadelphia, Pennsylvania, United States of America

*** Correspondence:**
Corresponding Author
dmeaney@seas.upenn.edu

Keywords: traumatic brain injury, neuronal networks, susceptibility, calcium imaging, connectivity, circuit scaling. (Min.5-Max. 8)

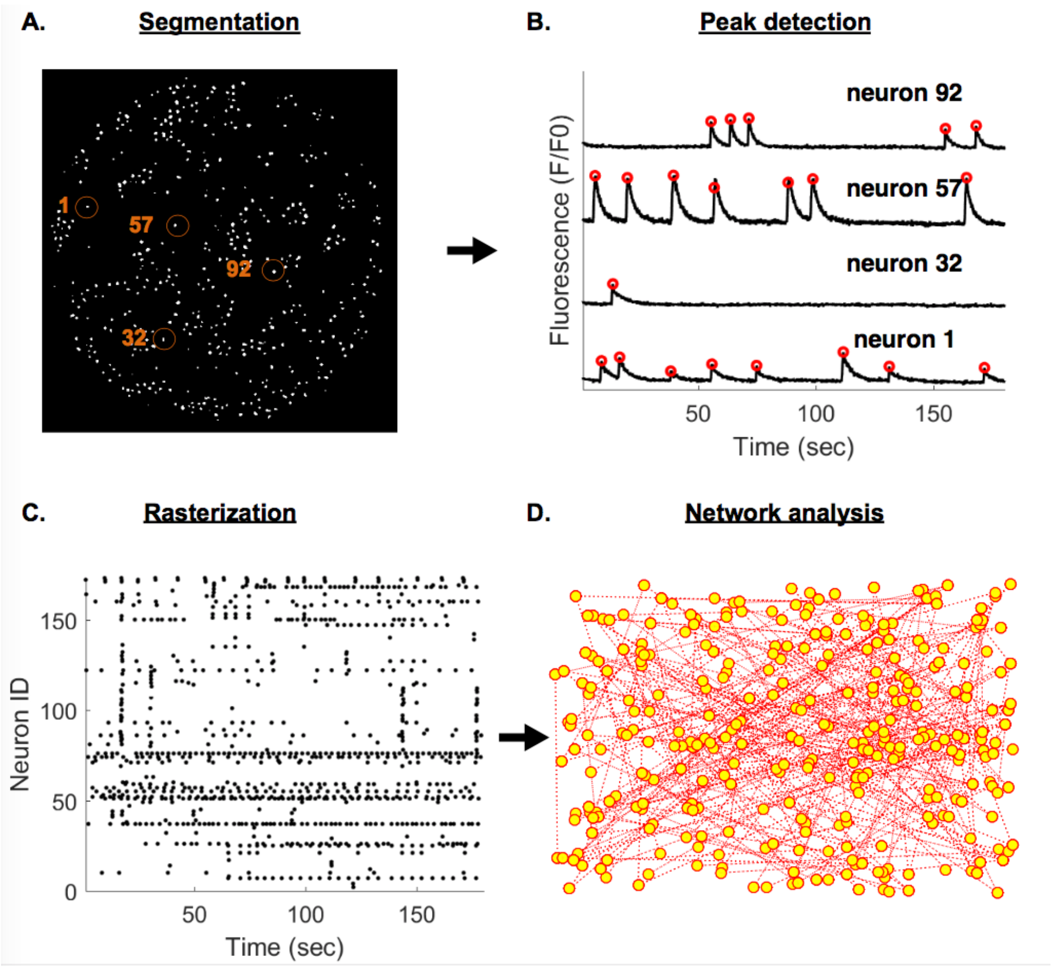

Supplementary Figure 1: Diagram of analysis pipeline. A. Manual segmentation of the maximum projection file for each .tiff stack of Ca^{2+} activity fluorescence yielded a binary mask where single neurons are represented as individual regions of interest (ROIs, orange circles). **B.** Single-cell Ca^{2+} transients were detected accurately using a MATLAB-based analysis system comparing brief segments of the fluorescence trace with a library of transient traces. **C.** Raster plots summarizing activity data from all cells within one island allow for quick visualization of activity patterns; each dot marks a detected transient. **D.** Functional connectivity networks constructed based on pairwise phase synchronization provide further information about the functional state of island circuits (yellow circles – nodes; red lines – binary pairwise functional connection).

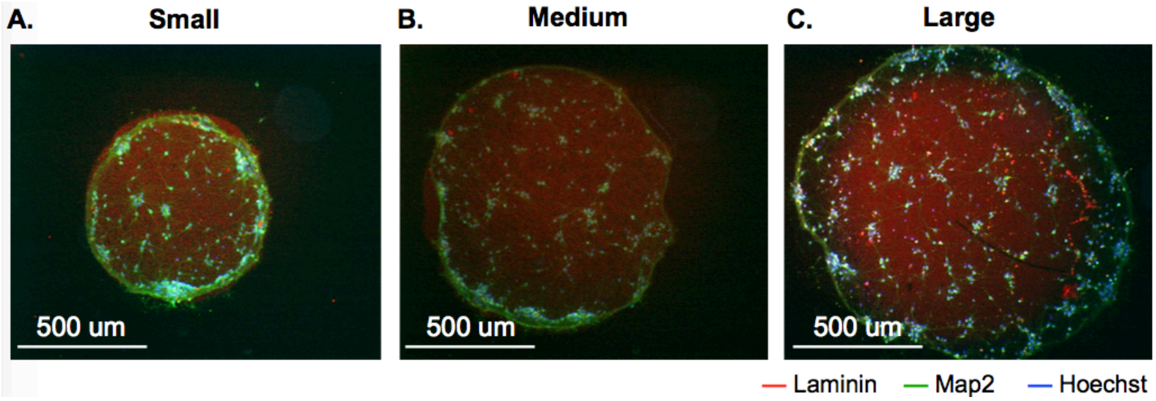

Supplementary Figure 2: Immunolabeling of cell distribution in neuronal islands. Neurons selectively attached and migrated onto restricted areas of Laminin and PDL coating (red, Rb~Laminin) in patterned islands of different diameters (small: 750um; medium: 1,000um; large: 1,300 um). By 14DIV, cell bodies were distributed evenly across the island surface as evidenced by the DNA-binding stain Hoechst (blue), and connected through a well-developed network of neuronal projections (green, Ms~MAP2).

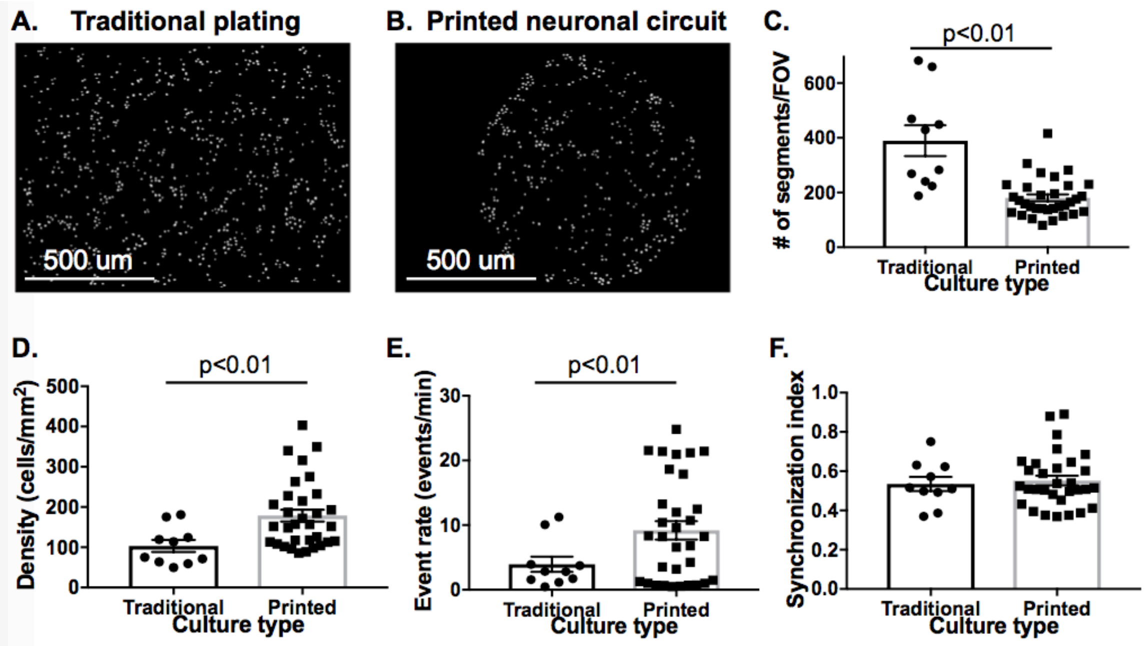

Supplementary Figure 3: Comparison between traditionally-plated dissociated cultures and neuronal islands. A-B. Segmentation masks corresponding to traditionally-plated dissociated culture and an island culture indicate overall even distribution of cells on both platforms by 10 DIV. **C-F.** Summary statistics from 10 traditional cultures and 32 neuronal islands reveal a higher number of cells identified in the traditional cultures **(C),** but an overall proportionately higher cellular density in islands **(D).** Functionally, activity rate, but not synchronization level, was elevated in islands compared to dissociated controls **(E-F).** Error bars indicate SEM.

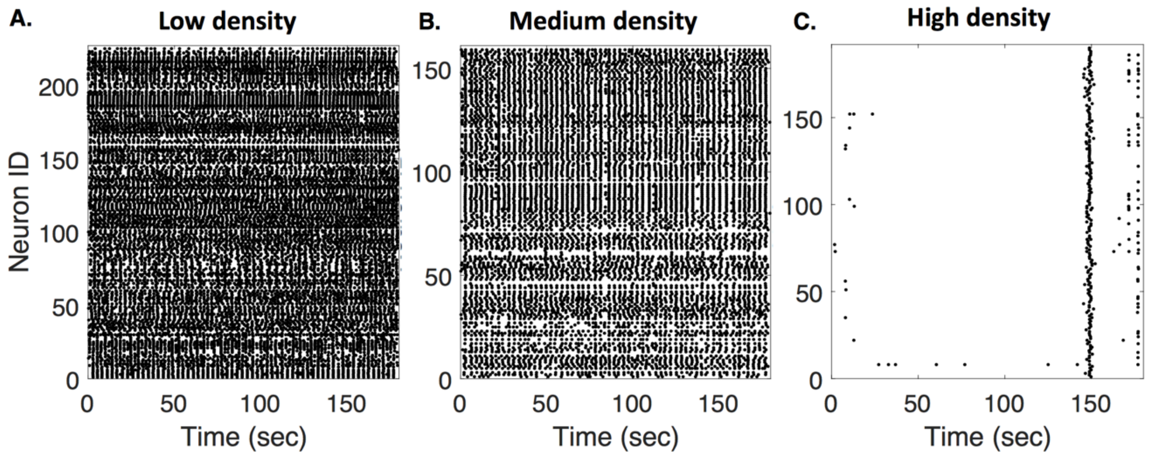

Supplementary Figure 4: Representative examples of activity patterns in neuronal islands of varying cellular densities. High rates of uncorrelated activity **(A)** gradually transitioned to highly synchronous, low-rate activity **(C)** as cellular density was increased from low (<175 cells/mm^2) to high (>250 cells/mm^2) values.
